# Confusoside from *Anneslea fragrans* Alleviates Acetaminophen-Induced Liver Injury in HepG2 via PI3K-CASP3 Signaling Pathway

**DOI:** 10.3390/molecules28041932

**Published:** 2023-02-17

**Authors:** Jing-Hao Zhao, Jing Li, Xiao-Yu Zhang, Shang Shi, Lin Wang, Ming-Long Yuan, Ya-Ping Liu, Yu-Dan Wang

**Affiliations:** 1 Key Laboratory of Chemistry in Ethnic Medicinal Resources, State Ethnic Affairs Commission and Ministry of Education, Yunnan Minzu University, Kunming 650500, China; 2 Department of Information, The First People’s Hospital of Yunnan, Kunming 650021, China; 3 The faculty of Food Science and Engineering, Kunming University of Science and Technology, Kunming 650500, China; 4 School of Chemistry and Environment, National and Local Joint Engineering Research Center for Green Preparation Technology of Biobased Materials, Yunnan Minzu University, Kunming 650500, China

**Keywords:** confusoside, dihydrochalcone glucoside, inflammation, apoptosis, liver injury

## Abstract

Confusoside (CF), a major chemical compound in the leaves of *Anneslea fragrans* Wall., is a dihydrochalcone glycoside with excellent antioxidant and anti-inflammatory effects. However, the hepatoprotective effect of CF has not been described. This study aimed to explore the hepatoprotective effect of CF against acetaminophen (APAP)-induced hepatic injury in HepG2 cells. First, the potential hepatoprotective effect mechanisms of CF were predicted by network pharmacology and were thought to involve reducing inflammation and inhibiting apoptosis. Target proteins (phosphatidylinositol3-kinase (PI3K) and caspase-3 (CASP3)) were found via molecular docking analysis. To verify the predicted results, an analysis of biological indicators was performed using commercial kits and Western blotting. The results showed that CF significantly decreased the levels of liver injury biomarkers (ALT, AST, and LDH), strongly inhibited the production of inflammatory cytokines (IL-1*β*, IL-6, and TNF-α) and the NO level via inhibiting the activation of the NF-*κ*B signaling pathway, and markedly regulated the expression levels of Bcl2, Bax, and cleaved-CASP3/9 proteins by activating the PI3K-CASP3 apoptosis pathway. The results demonstrated that CF has a therapeutic effect on APAP-induced liver injury by inhibiting intracellular inflammation and cell apoptosis, indicating that CF may be used as a potential reagent for the prevention and treatment of APAP-induced liver injury.

## 1. Introduction

The occurrence of liver-related diseases (e.g., hepatitis, cirrhosis, and liver cancer) has been increasing over the past two decades, and approximately 2 million people die from liver diseases globally every year [1]. The excessive intake of drugs and toxic substances can lead to liver injury, and acetaminophen (APAP) is one of the main factors related to liver damage. APAP is the most commonly used antipyretic and analgesic drug in clinical settings, and its use was especially high during the COVID-19 pandemic as a first-line drug for COVID-19 patients. However, the excessive and unreasonable reuse of APAP can cause serious liver damage in patients, which has become a concerning phenomenon [2].

APAP-induced liver injury has been used in many studies to explore the hepatoprotective effect of natural products. Excessive APAP metabolism in the body produces a toxic intermediate, NAPQI, which leads to inflammation and apoptosis [3,4]. This can cause abnormal liver cell death, resulting in severe liver damage. At present, the available clinical drugs for treating liver diseases are synthetic molecules with diverse side effects [5,6]. Though NAC is the only drug currently approved for the treatment of APAP overdose, the treatment window of NAC is narrow, and it cannot completely eliminate the damage caused by APAP [4,6]. Therefore, there is an urgent need for an effective hepatoprotective agent with low toxicity. The most direct and effective way to achieve this is to consider the main ingredients that protect the liver in medicinal and edible homologous plants.

Natural products are valuable resources for the development of functional food additives and pharmaceutical ingredients due to their complex structures and significant biological activities [2]. Dihydrochalcones, an important class of flavonoids, are formed by two phenolic rings connected by an open-chain three-carbon linkage with a C6-C3-C6 skeleton [7]. Many dihydrochalcones (i.e., phloretin, phlorizin, and trilobatin) are significant plant-derived natural products and are mainly found in apples and sweet tea [8,9]. They have exhibited excellent antioxidant effects and hepatoprotective activities [10,11,12,13]. The substituted location and type of glycoside moiety in dihydrochalcones influence the production of cytokines, therefore influencing their activities [14,15]. Research on dihydrochalcone glycosides is of importance for human health.

Confusoside (CF) is a dihydrochalcone glucoside, also known as davidigenin 4′-*O*-*β*-d-glucoside, which is mainly present in the plants of the *Anneslea* and *Symplocos* genera. The leaves of *Anneslea fragrans*, or “Pangpo Tea”, are traditionally used as an herbal tea for the treatment of liver and intestinal diseases [16,17,18]. They have antioxidant and anti-inflammatory effects [17,18,19]. Our studies found that CF is a major chemical compound in the leaves of *A. fragrans*, which may have beneficial antioxidant and anti-inflammatory effects [17,18,19], but whether CF has a hepatoprotective effect in liver disease has not yet been explored. In this paper, we were the first to investigate the protective effect of CF against liver injury induced by excessive APAP. The hepatoprotective effect of CF was investigated using an APAP-induced HepG2 cell damage model. The inhibitory effects of CF against cell inflammation and apoptosis were evaluated by enzyme-linked immunosorbent assay (ELISA), Western blotting (WB), and flow cytometry assays. The possible molecular mechanism was studied by a combination of network pharmacology, molecular docking, and Western blotting techniques. This paper may provide important information on a valuable hepatoprotective effect ingredient (CF).

## 2. Results

### 2.1. HPLC Analysis of CF

**Figure 1:**
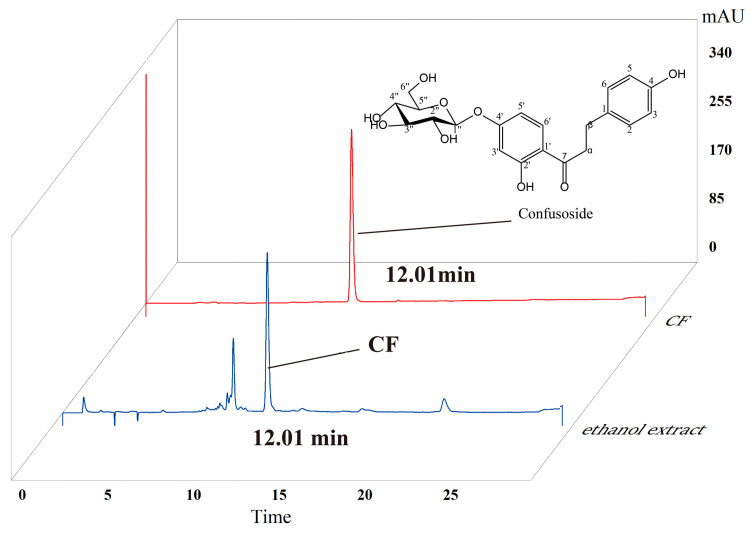
Chemical profiles of ethanol extract of *Anneslea fragrans* and confusoside (CF).

As shown in Figure 1, high-performance liquid chromatography (HPLC) analysis showed that the retention time of the main chemical compound in the ethanolic extract of *A. fragrans* leaves was observed at 12.01 min. This principal compound was separated by medium-pressure liquid chromatography (MPLC) column chromatography under the guidance of HPLC. The chemical structure of this compound was identified as confusoside (CF) by comparison of the 1D-NMR spectroscopic data with the reported data in the literature (Supplementary Figures S1 and S2) [17]. Its molecular formula was determined as C_21_H_24_O_9_ by the molecular ion at m/z 421.14 [M + H]^+^ in the ESIMS spectrum. Finally, the purity of CF was greater than 98% according to HPLC analysis, and the content of CF accounted for 28.12% of the ethanolic extract from *A. fragrans* (Figure 1).

### 2.2. Potential Targets of CF Treatment of Liver Injury

**Figure 2:**
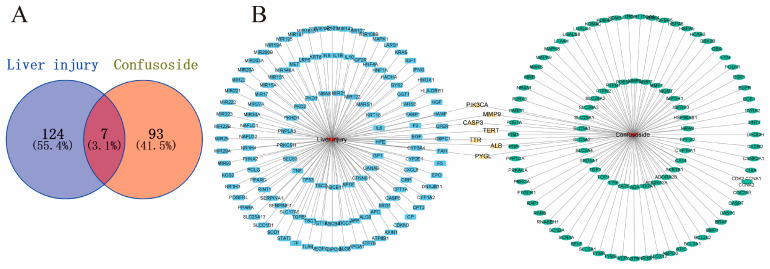
The Venn diagram of CF and liver injury targets (**A**); the target gene network (**B**).

The widespread use of network pharmacology has become a new approach to studying the mechanisms of compounds in the treatment of disease [20]. A total of 100 possible targets for CF in liver diseases were obtained from the Swiss Target Prediction. To identify relevant targets for liver injury, a total of 8447 relevant targets were screened from GeneCards. However, only 131 targets with correlation scores > 20 were selected for further investigation (Figure 2A) (Supplementary Table S1). Seven intersecting targets, PI3-kinase p110-alpha subunit (PI3KCA), matrix metalloproteinase 9 (MMP9), caspase-3 (CASP3), telomerase reverse transcriptase (TERT), transthyretin (TTR), albumin (ALB), and liver glycogen phosphorylase (PYGL), were found in the interaction network of “compound–target–disease” (Figure 2B).

### 2.3. GO Biological Process and KEGG Pathway Enrichment Analysis

**Figure 3:**
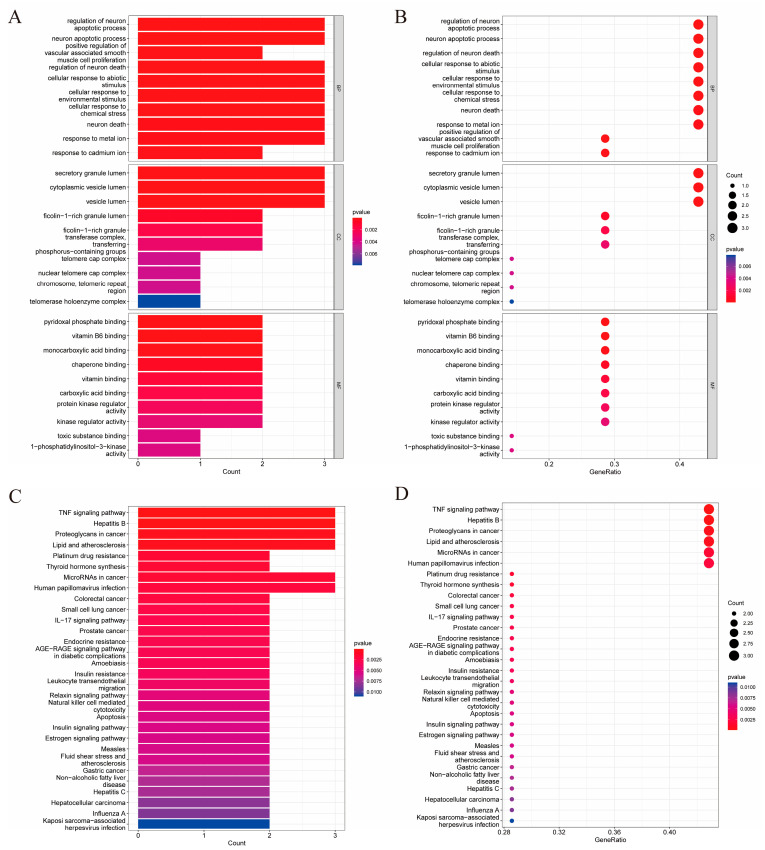
Network pharmacological analysis of the hepatoprotective activity of confusoside (CF). GO enrichment and KEGG pathway analysis of candidate targets. The top 10 significantly enriched GO terms in BP, CC, and MF (**A**). The enrichment analysis of the GO enrichment analysis (**B**). The top 30 pathways of the KEGG pathway analyses (**C**). The enrichment analysis of the KEGG signaling pathways (**D**). For C and E, x-axis represents count while y-axis illustrates the enriched terms with ID and name; for D and F, x-axis represents gene ratio (gene count/gene size) while y-axis illustrates the enriched terms with ID and name.

To explore the therapeutic mechanism of CF in liver injury, GO and KEGG pathway enrichment analyses were performed using Rx64 4.1.2. GO is used to divide the functions of genes into cellular component (CC), molecular function (MF), and biological process (BP) categories [21]. There were 341 BP, 21 CC, and 58 MF terms with a *p*-value ≤ 0.05. The top 10 terms in each of the BP, CC, and MF categories in the GO analysis are shown in Figure 3A. The analysis results are visually presented using bubble plots in Figure 3B. BP analysis showed that the relevant targets were mainly focused on apoptosis and chemical or environmental stress pathways. KEGG pathway enrichment analysis of involved targets was performed to further reveal the underlying mechanisms affecting the involved pathways of CF in liver injury. A total of 30 pathways were obtained from KEGG analysis for visualization according to their *p* values (Figure 3). Foremost among them was the TNF signaling pathway (HSA04668).

### 2.4. The Relationship of CF with Liver Injury Assessed by Molecular Docking

**Figure 4:**
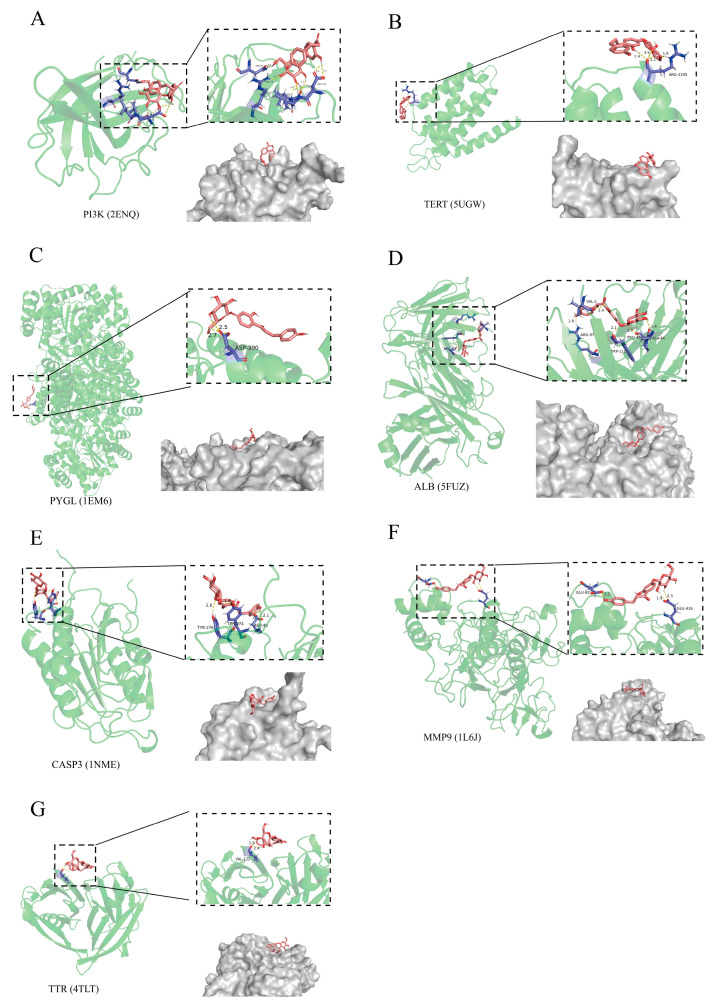
Molecular docking of confusoside (CF) to liver injury targeted genes. PI3-kinase p110-alpha subunit (PI3KCA) (**A**); telomerase reverse transcriptase (TERT) (**B**); liver glycogen phosphorylase (PYGL) (**C**); albumin (ALB) (**D**); caspase-3 (CASP3) (**E**); matrix metalloproteinase 9 (MMP9) (**F**); transthyretin (TTR) (**G**).

For molecular docking, a binding energy of <0 between compounds and proteins can be considered indicative of binding in a spontaneous manner, whereas a binding energy of <−7 kJ/mol is considered indicative of binding in a more stable manner. A lower binding energy means that the binding is more stable [22]. The molecule docking binding energies of CF with the vital targets were PI3KCA (2ENQ) = −7.15 kJ/mol, TERT (4TLT) = −1.30 kJ /mol, PYGL (1EM6) = −5.82 kJ /mol, ALB (5FUZ) = −13.01 kJ/mol, CASP3 (1NME) = −9.83 kJ/mol, MMP9 (1L6J) = −0.71 kJ/mol, and TTR (4TLT) = −1.29 kJ/mol. The binding energy between CF and the target proteins was assessed via hydrogen bonding, bond lengths, CF and the amino acid residues, and 3D interactions (Figure 4). The docking results showed that CF had a strong binding ability with CASP3, PI3K, and ALB. The liver synthesizes ALB, an indicator for evaluating liver damage, but this cannot regulate liver health and cannot be used as a target for liver protection. Therefore, the key targets of CF liver protection may be PI3KCA and CASP3-related proteins.

### 2.5. CF Reduced APAP-Induced Injury of Hepg2 Cells and Improved Their Viability

**Figure 5:**
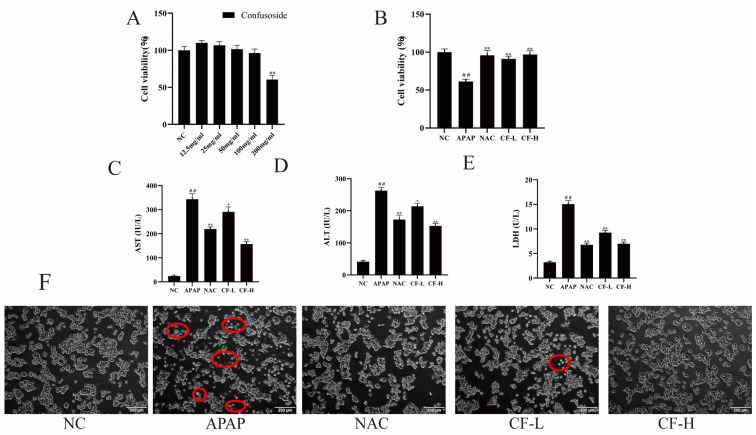
The protective effect of confusoside (CF) on liver injury in APAP-induced HepG2 cells. Cell viability of CF-treated HepG2 cells (**A**); cell viability of CF-treated APAP-induced HepG2 cells (**B**); the levels of aspartate aminotransferase (AST) (**C**), alanine aminotransferase: glutamate pyruvic transaminase (ALT) (**D**), and lactate dehydrogenase (LDH) (**E**) and cell morphological changes (**F**) in APAP-induced HepG2 cells. The red circles indicate cells with significant damage. The mean ± SD is presented. ## *p* < 0.01 vs. group NC; * *p* < 0.05 and ** *p* < 0.01 vs. group APAP.

Predicted results are not absolutely accurate and need to be further verified by experiments. The cell survival rate decreased with increasing concentration of CF. The cell viability was below 90% at a concentration of 200 μg/mL and above 90% at a concentration of 100 μg/mL (Figure 5A). Therefore, the concentrations of 50 and 100 μg/mL were chosen for subsequent experiments.

The therapeutic effects of the CF-L and CF-H groups on APAP-induced HepG2 cells were evaluated by cell viability and important biomarkers of liver injury (ALT, AST, and LDH). The cell survival rate of the APAP group (60%) was significantly lower than that of the NC group (natural control group) (100%) (*p* < 0.01) (Figure 5B), which indicated that the APAP-induced HepG2 injury model was successfully established [23]. Furthermore, the cell viability of CF-L and CF-H groups significantly increased to more than 90%, close to that of the NC group. ALT, AST, and LDH are important biomarkers of liver injury [24,25,26]. Both the CF-L and CF-H groups exhibited significantly decreased levels of liver injury biomarkers (ALT, AST, and LDH) in HepG2 cells in comparison with the APAP group. The levels of the three biomarkers were lower in the CF-H group (ALT: 152.52 ± 7.87 IU/L; AST: 156.94 ± 9.13 IU/L; LDH: 7.01 ± 0.31 U/L) than in the NAC group (ALT: 172.33 ± 13.96 IU/L; AST:219.27 ± 8.55 IU/L; LDH: 6.78 ± 0.33 IU/L) (positive control) (*p* < 0.01), which suggested the inhibitory effects of the CF-H group was superior to those of the NAC group (Figure 5C–E). These results confirmed that CF has a significant hepatoprotective effect against acetaminophen (APAP)-induced hepatic injury.

Furthermore, the cell morphology was assessed using an inverted microscope (Figure 5F). Phase contrast microscopy uses the principle of light interference to determine the condition of cells based on their brightness. Damaged cells exhibit significant organelle shrinkage, which can highlight the cells in phase contrast microscopy. Compared to the NC group, there was a significant degree of cell death in the APAP group. The cell death of the CF-L and CF-H groups was significantly improved compared with that of the APAP group, indicating that CF had a mitigating effect on cell death. The effect of the CF-H group was more pronounced than that of the CF-L group, and there was a dose-dependent relationship. These findings suggested CF can reduce the cell damage caused by APAP in HepG2 cells.

### 2.6. Inhibitory Effect of CF on APAP-Induced Inflammation in HepG2 Cells

**Figure 6:**
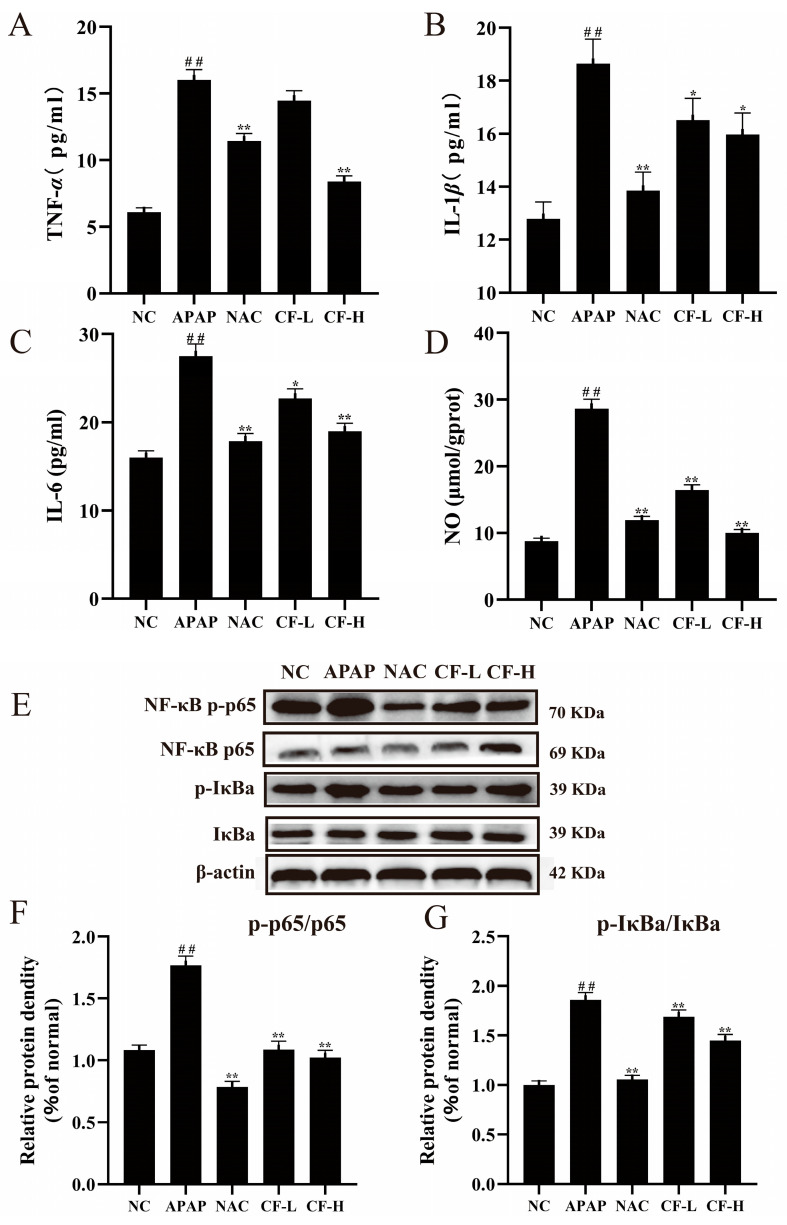
Anti-inflammatory effects of confusoside (CF) on APAP-induced HepG2 cells. The expression levels of inflammatory cytokines tumor necrosis factor-*α* (TNF-*α*) (**A**), interleukin-1*β* (IL-1*β*) (**B**), interleukin-6 (IL-6) (**C**), and nitric oxide (NO) (**D**). Immunoblot bands of hepatic protein expression levels of NF-*κ*B p-p65, NF-*κ*B p65, p-I*κ*B*α*, and I*κ*B*α* (**E**); quantification results for NF-*κ*B p-p65/NF-*κ*B p65 (**F**) and p-I*κ*B*α*/I*κ*B*α* (**G**). The mean ± SD is presented. ## *p* < 0.01 vs. group NC; * *p* < 0.05 and ** *p* < 0.01 vs. group APAP.

The occurrence of inflammation can lead to apoptosis and liver damage [27]. The inflammatory cytokine and NO levels were used to examine the potential mechanism of CF in APAP-induced injury of HepG2 cells. The APAP group had higher levels of pro-inflammatory cytokines (IL-6, IL-1*β*, TNF-*α*) and NO levels (*p* < 0.01) compared with the NC group (Figure 6A–D), implying that APAP causes cellular inflammatory responses. For the CF-L group, there was a significant decrease in NO levels (*p* < 0.01) and a weak effect for pro-inflammatory cytokines IL-1*β* and IL-6 (*p* < 0.05). Compared to the APAP group, treatment with NAC significantly reduced the levels of TNF-*α* (11.43 ± 0.53 pg/mL) and NO (11.91 ± 0.61 μmol/gprot) (*p* < 0.01) and also strongly decreased the levels of inflammatory cytokines IL-6 (17.85 ± 0.87 pg/mL) and IL-1*β* (15.98 ± 0.81 pg/mL) (*p* < 0.05). Surprisingly, the CF-H group had lower levels of TNF-*α* (8.38 ± 0.41 pg/mL) and NO (10.1 ± 0.43 μmol/gprot) (*p* < 0.01) than the NAC group, and the levels of IL-6 (18.96 ± 0.87 pg/mL) and IL-1*β* (15.98 ± 0.81 pg/mL) were equal to those of the NAC group (Figure 6A–D). The aforementioned data suggested that the production of pro-inflammatory cytokines and NO could have been significantly inhibited in the CF-L and CF-H groups.

Based on the results of GO and KEGG analyses, it was necessary to investigate the proteins associated with the TNF inflammatory pathways. The p65-NF-*κ*B is a key regulatory protein in the inflammatory pathway, and its expression level reflects intracellular inflammation [28]. The expression levels of p-p65/p65 and p-I*κ*Bα/I*κ*Bα in the CF-H and CF-L groups were significantly higher than those of the APAP group (*p* < 0.01) (Figure 6E–G), which suggested that inflammatory damage in APAP-induced HepG2 cells was remarkably inhibited via the NF-*κ*B signaling pathway in the CF group.

### 2.7. Inhibitory Effect of CF against APAP-Induced Apoptosis in HepG2 Cells

As shown in Figure 6A, cell apoptosis was significantly increased in the APAP group compared to the NC group (*p* < 0.01). Compared with the APAP group, apoptosis was significantly reduced in the drug-treated cells, both in NAC and CF (*p* < 0.01). With increasing CF dose, the inhibition of apoptosis in the group CF-H was superior to that in the CF-L group and comparable to that in the NAC group.

**Figure 7:**
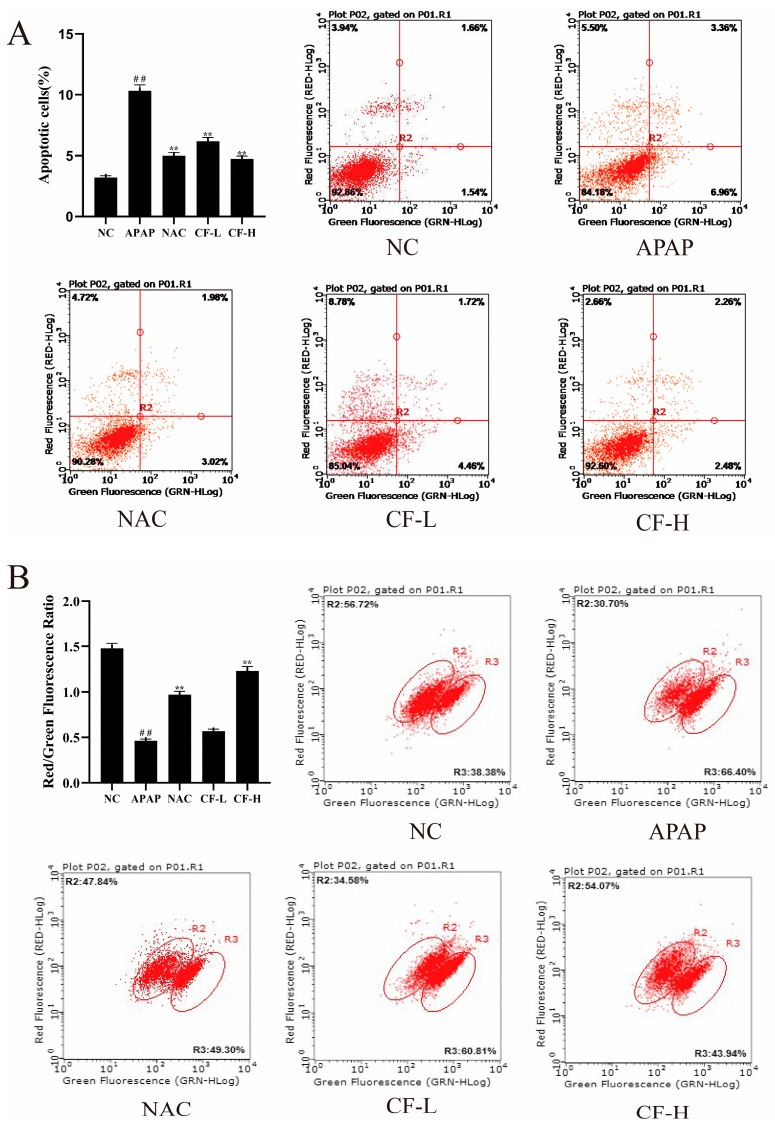
Effects of confusoside (CF) on cell apoptosis (**A**) and mitochondrial membrane potential (MMP) (**B**) in the APAP-induced HepG2 cells. The mean ± SD is presented. ## *p* < 0.01 vs. group NC and ** *p* < 0.01 vs. group APAP.

Fluorescent proteins were used to measure the degree of cell apoptosis. When there is red fluorescence, the mitochondrial membrane potential is relatively normal and the cell is in a normal state; when there is green fluorescence, the mitochondrial membrane potential has decreased and the cell is in the early stages of apoptosis [4]. As shown in Figure 7A, cell apoptosis was significantly increased in the APAP group (10.32 ± 0.61) compared to the NC group (3.2 ± 0.11) (*p* < 0.01). Compared with the APAP group, both the CF-H (4.74 ± 0.21) and CF-L (6.18 ± 0.29) groups showed significantly reduced cell apoptosis (*p* < 0.01), equal to that of the NAC group (5.01 ± 0.26). The CF-H group was more inhibited against APAP-induced HepG2 apoptosis than the CF-L group and showed a dose-dependent relationship. After APAP treatment, the proportion of green fluorescence significantly increased, and accordingly, the proportion of red fluorescence decreased in the cells (*p* < 0.01) (Figure 7B). The proportion of red fluorescence of the CF-H (1.23 ± 0.04) group strongly increased compared with that of the APAP group (0.46 ± 0.02) (*p* < 0.01), which suggested CF can decrease cell apoptosis in APAP-induced HepG2 cells. The protective effects of the CF-H group were superior to those of the NAC group (positive control) and were equal to those of the NC group (black control) (Figure 7B).

**Figure 8:**
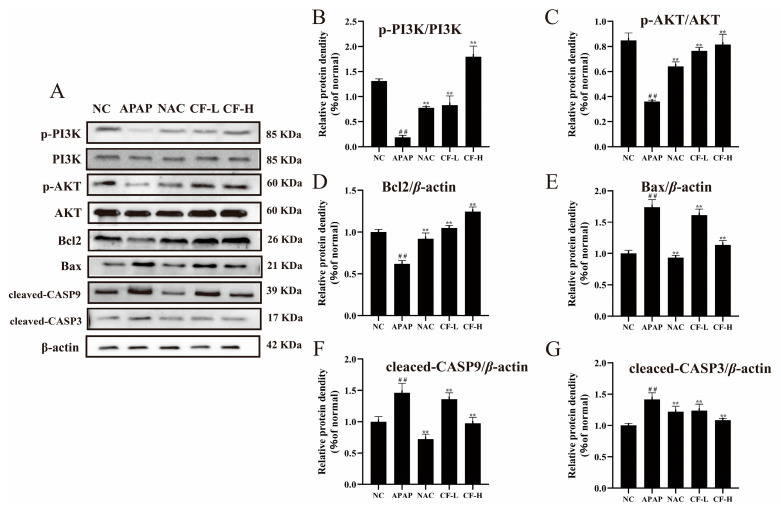
Anti-apoptotic effects of confusoside (CF) on APAP-induced HepG2 cells. Immunoblot bands of hepatic protein expression levels of phospho-PI3K (p-PI3K), phosphatidylinositol 3-kinase (PI3K), phospho-Akt (p-Akt), protein kinase B (Akt), B-cell lymphoma-2 (Bcl2), Bcl2-associated X (Bax), cleaved-caspase-3, and cleaved-caspase-9 as determined by Western blotting analysis (**A**). Quantification results for p-PI3K/PI3K (**B**), p-Akt/Akt (**C**), Bcl2 (**D**), Bax (**E**), cleaved-caspase-9 (**F**), and cleaved-caspase-3 (**G**). The mean ± SD is presented. ## *p* < 0.01 vs. group NC and ** *p* < 0.01 vs. group APAP.

Western blotting analysis further verified the PI3K/Akt and CASP3 apoptosis pathways in APAP-induced damage in HepG2 cells (Figure 8). The CF group showed significantly increased expression levels of p-PI3K/PI3K, p-Akt/Akt, and Bcl2 and decreased expression levels of Bax, cleaved-CASP3, and cleaved-CASP9 compared with the APAP group (*p* < 0.01) (Figure 8). Notably, the protein expression of the CF-L group was comparable to that of the NAC group, while the protein expression of the CF-H group (p-PI3K/PI3K: 1.79 ± 0.21; p-Akt/Akt: 0.82 ± 0.08; Bcl2: 1.24 ± 0.06; Bax: 1.13 ± 0.07; cleaved-CASP3: 1.08 ± 0.02; cleaved-CASP9: 0.97 ± 0.09) was superior to that of the NAC group (p-PI3K/PI3K: 0.78 ± 0.03; p-Akt/Akt: 0.64 ± 0.04; Bcl2: 0.92 ± 0.07; Bax: 0.93 ± 0.04; cleaved-CASP3: 1.22 ± 0.09; cleaved-CASP9: 0.72 ± 0.08). These results confirmed that CF has a significant therapeutic effect in APAP-induced liver injury.

## 3. Discussion

It is important to select appropriate cell models to validate the predicted results and investigate the mechanism by which CF prevents APAP-induced liver injury. HepG2 cells are a typical hepatocyte model for studying pharmacological toxicity [25]. The activities of ALT, AST, and LDH are important biological indicators for evaluating liver injury [24,26]. Observation of cell viability and morphology is the most direct way to evaluate cell damage. Therefore, the therapeutic effect of CF was evaluated by measuring the activities of ALT, AST, and LDH on APAP-induced liver cell damage. The present experimental results were clearly able to reflect the preventive effect of CF against damage in APAP-induced HepG2 cells.

Liver injury is usually accompanied by an inflammatory response, which is regulated by the NF-*κ*B pathway. In the cytoplasm, NF-*κ*B is normally combined with its inhibitor I*κ*B*α*, in a silent and inactive state. As I*κ*B*α* degrades, free NF-*κ*B subunits will migrate into the cell nucleus, resulting in the activation of the NF-*κ*B pathway to promote cell cytokine expression [29]. The TNF pathway regulates many pathways, among which the NF-*κ*B pathway is the main downstream pathway. Proinflammatory cytokine TNF-*α* is known as one of the earliest inducers in liver injury, and it can also degrade I*κ*B*α* to activate the NF-*κ*B pathway, stimulating the production and secretion of some cytokines, including IL-1*β* and IL-6 [30]. After activation by APAP, NF-*κ*B is translocated to the nucleus and regulates the expression of related inflammatory cytokines, thereby leading to inflammatory damage [31]. Similar results were also observed in this study, where CF effectively suppressed inflammatory responses by inhibiting the activation of the NF-*κ*B pathway and reducing the production of TNF-*α*, IL-1*β*, and IL-6.

According to the results of GO and KEGG analysis, inhibition of apoptosis is also an important mechanism of CF in the treatment of liver damage caused by APAP. Flow cytometry was used to analyze the change in mitochondrial membrane potential, which is a marker of the early apoptosis of cells. As an important anti-apoptotic signaling pathway, the PI3K/Atk signaling pathway exerts its effect by activating the Bcl2 protein and inhibiting CASP3 activity [32]. In addition, as an upstream pathway, the NF-*κ*B-regulated inflammatory pathway also can regulate the protein expression levels of Bcl2 and CASP. Due to the potential relationship between PI3K, CASP3, and CF predicted by molecular docking, WB analysis was performed to determine the related proteins in the PI3K-CASP3 apoptosis pathway, further verifying that the PI3K/Akt/CASP3 signaling pathways are the key pathways in APAP-induced HepG2 cell damage.

Therefore, the liver-protective mechanism of CF against APAP-induced liver injury was assessed by predicting and analyzing signaling pathways and then verified by the experimental results. CF can effectively suppress the inflammatory response in APAP-induced HepG2 cells through the activation of the NF-κB pathway and reduction in levels of TNF-α, IL-1β, and IL-6 proteins. Additionally, CF can interact with PI3K/Akt to regulate downstream Bcl2/Bax and CASP3 expression levels, which effectively alleviates cell apoptosis. CF, therefore, achieved a therapeutic effect in the liver via inhibiting the NF-κB-regulated inflammatory response and PI3K/Akt-regulated apoptosis.

## 4. Material and Methods

### 4.1. Chemicals and Reagents

Fetal bovine serum (FBS), Dulbecco’s modified Eagle’s medium (DMEM), phosphate-buffered saline (PBS), penicillin, and streptomycin were obtained from Gibco (Grand Island, NY). 3-(4,5-dimethylthiazol-2-yl)-2,5-diphenyltetrazolium bromide (MTT), 2′,7′-dichlorofluorescindiacetate (DCFH-DA), calcium ion fluorescent probe Fluo-4 AM, N-acetyl-cysteine (NAC), and 5,5′,6,6′-tetrachloro-1,1′,3,3′-tetraethylimidacarbocyanine iodide (JC-1) were purchased from Sigma-Aldrich (Shanghai, China). Antibodies (*β*-actin; PI3K, phosphatidylinositol 3-kinase; p-PI3K, phospho-PI3K; AKT, protein kinase B; p-AKT, phospho-AKT; NF-*κ*B p65; p-NF-*κ*B p65; I*κ*B*α*, recombinant inhibitory subunit of NF-*κ*B alpha; p-I*κ*B*α*; Bcl2, B-cell lymphoma-2; Bax, Bcl2-associated) were purchased from ABclonal (Wuhan, China). Other antibodies (cleaved-CASP3, cleaved cysteinyl aspartate specific proteinase-3; cleaved-CASP9, cleaved cysteinyl aspartate specific proteinase-9) were purchased from Abcam (Shanghai, China). ELISA kits for interleukin-1*β* (IL-1*β*), tumor necrosis factor-*α* (TNF-*α*), and interleukin-6 (IL-6) were obtained from MultiSciences (Lianke) Biotech (Hangzhou, China). Biochemical detection kits (ALT, alanine aminotransferase; AST, aspartate transaminase; LDH, lactate dehydrogenase; NO, nitric oxide) were purchased from the Nanjing Jiancheng Bioengineering Institute (Nanjing, China). Other reagents used were of analytical grade and purchased from Tianjin Fengchuan Chemical Reagent (Tianjin, China).

### 4.2. Preparation of CF from Leaves of A. fragrans

The CF was extracted, isolated, and purified from *A. fragrans* leaves, which were collected from Lincang city, China, in July 2020. A voucher specimen (number Cheng-20190514-01) was deposited in the Faculty of Food Science and Technology [19]. The air-dried leaves (50 g) were soaked in 500 mL of 80% ethanol solution, followed by ultrasound-assisted extraction (SB-5200D 360W, Ningbo xinzhi, Ningbo, China) three times (30 min per time). The extract was mixed and centrifuged at 1500× *g* for 10 min after the extraction process was finished. A vacuum-drying lyophilizer (Alpha 1-2 LD plus, Christ, Germany) was used to acquire the dried extract.

The extract was analyzed using high-performance liquid chromatography according to our previously reported method [17]. The chemical profiles of ethanol extract and CF were determined using an Agilent 1260 high-performance liquid chromatography (HPLC) system (Agilent Technologies Co. Ltd., Palo Alto, CA, USA) and an Agilent ZORBAX SB C18 column (5 μm, 250 mm × 4.6 mm). Acidified ultrapure water (0.1% formic acid) was used as mobile phase A, and acetonitrile was used as mobile phase B. The gradient conditions were as follows: 0 min (10% B), 0–5 min (30% B), 5–25 (50% B). The flow rate was set to 1.0 mL/min, and the injection volume was 10 μL. The temperature was maintained at 35 °C, and the detector wavelength was 254 nm.

The extract was subjected to a C_18_ column and isolated using a medium-pressure liquid chromatography (MPLC) system with a gradient solvent system of CH_3_OH-H_2_O (20:80, 40:60, 80:20, 10:0, *v*/*v*). After HPLC analysis, six fractions (Fr. A-F) were obtained. Fr. B was further purified with CH_3_OH-H_2_O (3:7, *v*/*v*) using a preparative HPLC system (Agilent 1260 liquid chromatograph) with a Zorbax SB-C_18_ column (21.2 mm × 250 mm, Agilent Technologies Co. Ltd., Palo Alto, CA, USA) to yield CF. The purity of CF was determined as > 98% using HPLC analysis.

### 4.3. Network Pharmacology Analysis of the Effect of CF on Liver Injury

The screening of targets against liver injury hits and the relative candidate targets of CF were identified based on databases. The relevant chemical information and SMILES structure of CF were searched through the PubChem database, and the chemical structure of CF was drawn using ChemDraw 19.0 software. Potential targets were predicted through Swiss Target Prediction (http://www.swisstargetprediction.ch/) (accessed on 3 March 2022) by using the chemical structure of CF and SMILES as search criteria. The potential targets related to liver injury were screened from GeneCards (https://www.genecards.org/) (accessed on 3 March 2022) [33]. The obtained targets were analyzed, and then those with correlation scores greater than 20 were selected for further study. The overlapping parts of the targets related to CF and liver injury obtained from the two searches were considered CF targets for liver injury and used for further analysis. All the targets were identified by their standardized ID in the UniProt database (https://www.uniprot.org/) (accessed on 3 March 2022) [34].

The common proteins between CF and liver injury were determined using VENNY (https://bioinfogp.cnb.csic.es/tools/venny/) (accessed on 4 March 2022) and derived from Venn diagrams. The “compound–target–disease” interrelationship was visualized using Cytoscape 3.9.1 software [35].

In order to further explore the mechanism of CF in liver injury, the vital targets were analyzed by Gene Ontology (GO) enrichment analysis and Kyoto Encyclopedia of Genes and Genomes (KEGG) enrichment analysis [36]. GO enrichment analysis and KEGG pathway enrichment of the significant clusters were analyzed using Cluster Profiler in Rx64 4.1.2 software [21,37,38]. Statistical significance was demonstrated by the significance threshold of *p* < 0.05 for the functional enrichment analysis. Bubble charts and histograms were drawn using the ggplot2 package Rx64 4.1.2.

### 4.4. Molecular Docking Analysis of CF on Liver Injury

Molecular docking of the obtained core targets was used to evaluate their binding potential to CF. Autodock Vina 1.5.6 software was used to complete the molecular docking [39,40]. The 3D structures of CF and targeted proteins were downloaded from PubChem and RCSB PDB databases (http://www.rcsb.org/) (accessed on 15 April 2022), respectively [41]. After removing the water molecules and ligands using PyMol (v2.5.0), the obtained protein structures were imported into AutoDockTools (v1.5.6) to define active pockets for docking of protein molecules. The calculated binding energy was used to assess the binding capacity and stability.

### 4.5. Cell Culture

Hepatoma HepG2 cells were purchased from Kunming cell bank, CAS, and were cultured in DMEM supplemented with 10% fetal bovine serum (FBS) and 1% antibiotics (100 U/mL penicillin and 100 μg/mL streptomycin) in a humidified atmosphere containing 5% CO_2_ at 37 °C.

### 4.6. Cell Culture

MTT assay was used to assess the cytotoxicity and protective effect of the drug in HepG2 cells [42]. HepG2 Cells (2.0 × 10^4^ cells per well) were cultured in 96-well culture plates with 200 μL of DMEM for 24 h. After that, the cells were treated with 200 μL of CF solution of different concentrations (0,12.5, 25, 50, 100, and 200 μg/mL) and then incubated for 20 h. Subsequently, the cells were treated with 0.5 mg/mL MTT for 4 h, and dimethyl sulfoxide (DMSO) was added to dissolve the purple formazan crystals. Finally, the absorbance was detected at 570 nm using a Spectra Max M5 microplate reader (Molecular Device, San Jose, CA, USA).

HepG2 cell damage was induced with 10 mM APAP according to a previously reported method [31]. According to the experimental protocol, the cells were divided into five groups: NC group (natural control group), APAP group (10 mM APAP), NAC group (10 mM APAP with 100 μg/mL NAC), CF-L group (10 mM APAP with 50 μg/mL CF), and CF-H group (10 mM APAP with 100 μg/mL CF). The cell viability of each group after drug treatment was detected to judge the protective effect on cells. Then, cell morphology was observed in phase contrast mode using an inverted microscope.

### 4.7. Analysis of Biochemical Indicators in APAP-Induced HepG2 Cells

After the cell supernatant was collected, the ALT, AST, LDH, and NO levels were measured using commercial assay kits according to the manufacturer’s instructions. The levels of cytokines interleukin-1*β* (IL-1*β*), tumor necrosis factor-α (TNF-*α*), and interleukin-6 (IL-6) were determined using an ELISA kit.

### 4.8. Determination of Cell Apoptosis

A human annexin VFITC(AV)/PI apoptosis kit was used, as in our previously reported method. Briefly, HepG2 cells were inoculated in 12-well plates at a density of 2.0 × 10^5^ cells per well. After the cell incubation with CF, the cells were washed three times with pre-cooled PBS and then collected in 1.5 mL Eppendorf tubes after centrifugation at 1500× *g*. Then, 100 μL of buffer and 5 μL of Annexin V-FITC and PI were added to the cells and reacted for 10 min in a dark environment. Finally, apoptosis of cells was detected by flow cytometry (Guava easy Cyte 6-2 L, Millipore, Billerica, MA, USA).

### 4.9. Detection of Mitochondrial Membrane Potential (MMP)

The MMP assay of HepG2 cells was undertaken using a previously developed method [4]. JC-1 was used as a probe to detect MMP changes and early apoptosis. Cells were administered in groups in 12-well plates, collected 24 h after APAP treatment, and then washed twice with precooled PBS. The cells were then stained with JC-1 solution for 30 min at 37 °C. Finally, the intracellular MMP was detected by flow cytometry at a maximum excitation wavelength of 527 nm and maximum emission wavelength of 590 nm.

### 4.10. Western Blotting Analysis

The APAP-treated cells were lysed in RIPA with protease and phosphatase inhibitors for 30 min. The cells were centrifuged at 9000× *g* for 10 min at 4 °C and the supernatant was collected. The total protein content of the supernatant was determined using a BCA kit. After the BCA assay, the supernatant was fixed in 5 × loading buffer in boiling water for 10 min. The proteins were separated using SDS-PAGE gels and then transferred onto PVDF membranes. After 1 h of blocking with 5% nonfat milk, the membranes were incubated with appropriate primary antibodies overnight at 4 °C. Subsequently, the membranes were washed two times with TBST and hybridized with horseradish peroxidase-conjugated anti-rabbit or anti-goat immunoglobulin IgG secondary antibodies for 2 h [43]. After washing with TBST three times, the protein bands were visualized by an imaging system (VILBER Fusion FX7, Vilber Lourmat, Marne-la-Vallee, France) using enhanced chemiluminescence detection reagents. The band intensities were quantified using Image J gel analysis software.

### 4.11. Statistical Analysis

Statistical analysis was performed using Origin 2021 and included one-way analysis of variance (ANOVA) followed by Tukey’s test to examine significant differences. All the experiments were repeated three times. The results were visualized using GraphPad Prism8. *p* < 0.05 and *p* < 0.01 were considered statistically significant. The data were reported as the mean ± SD.

## 5. Conclusions

In conclusion, this study was the first to investigate the protective effect of CF against liver injury induced by excessive APAP. Network pharmacology predictions combined with cellular experiments revealed that the potential molecular mechanisms of CF treatment for liver injury may involve reducing inflammation and inhibiting apoptosis. Changes in inflammatory markers in HepG2 cells after CF administration and a decrease in apoptosis verified this possibility. In addition, the prediction that CF reduces liver injury through the PI3K-CASP3 and NF-*κ*B-I*κ*Bα pathway was also validated by Western blotting analysis. Based on the above, CF has great potential for the prevention and treatment of APAP-induced liver injury and could be further investigated as a possible functional compound. This work provides information and suggestions regarding the hepatoprotective effects of dihydrochalcones and their glycosides.

## Data Availability

Data will be made available on request.

## Supplementary Materials

The following supporting information can be downloaded at: https://www.mdpi.com/article/10.3390/molecules28041932/s1, Figure S1: The ^1^H NMR spectra of confusoside; Figure S2: The ^13^C and DEPT NMR spectra of confusoside; Table S1: The correlated targes of liver injury and confusoside.
