# Supplementary material for: Confusoside from Anneslea fragrans Alleviates Acetaminophen-Induced Liver Injury in HepG2 via PI3K-CASP3 Signaling Pathway
Source: Molecules. 2023 Feb 17;28(4):1932. doi: 10.3390/molecules28041932 (PMC9964309; doi:10.3390/molecules28041932)

Confusoside from *Anneslea fragrans* Alleviates Acetaminophen-Induced Liver Injury in HepG2 via PI3K-CASP3 Signaling Pathway

Contents of Supporting Information

No.	Contents	Page
1	Figure S1 The ^{1}H NMR spectra of confusoside	3
2	Figure S2 The ^{13}C and DEPT NMR spectra of confusoside	4

Figure S1 The ^{1}H NMR spectra of **CF**

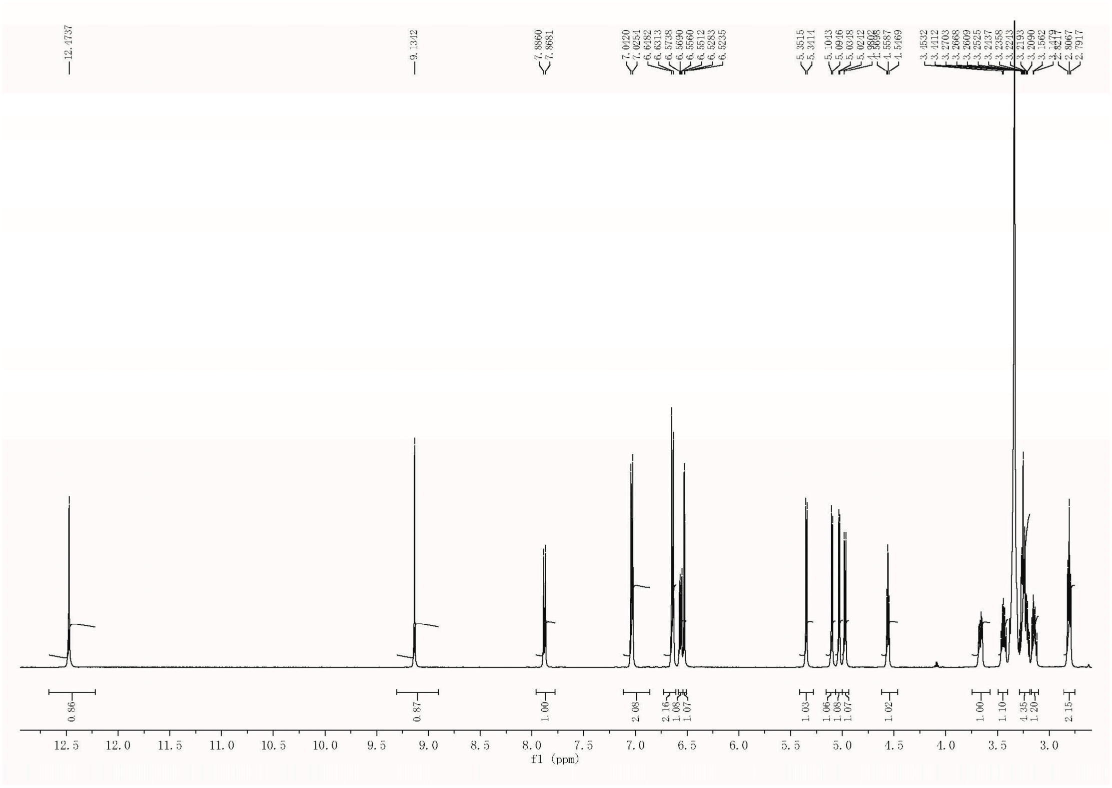

Figure S2 The ^{13}C and DEPT NMR spectra of **CF**

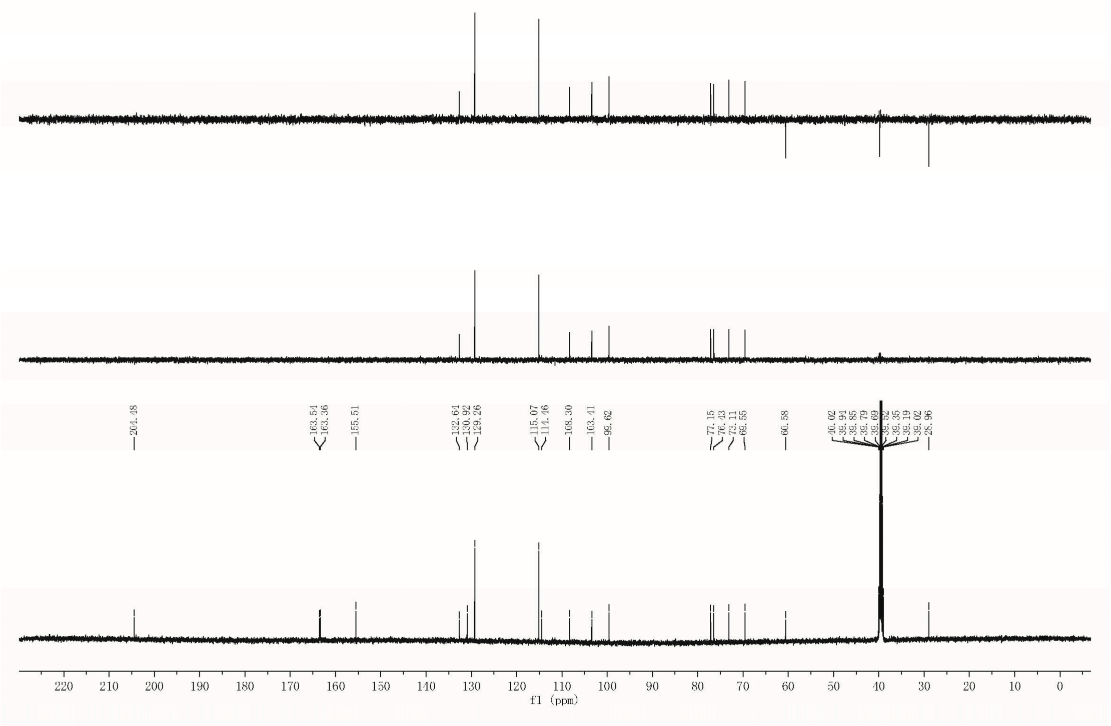

Supplement: Supplementary file 1 [file molecules-28-01932-s001.zip › Supplementary Figure.pdf]
