# Supplementary material for: Confusoside from Anneslea fragrans Alleviates Acetaminophen-Induced Liver Injury in HepG2 via PI3K-CASP3 Signaling Pathway
Source: Molecules. 2023 Feb 17;28(4):1932. doi: 10.3390/molecules28041932 (PMC9964309; doi:10.3390/molecules28041932)
Supplement: Supplementary file 1 [file molecules-28-01932-s001.zip › Supplementary Table.pdf]

# Confusoside from Anneslea fragrans Alleviates Acetaminophen-Induced Liver Injury in HepG2 via PI3K-CASP3 Signaling Pathway

# Contents of Supporting Information


| No. | Contents | Page |
|---|---|---|
| 1 | Table S1 The correlated targes of liver injury and confusoside | 3-6 |

| name | Gene | name | Gene |
|---|---|---|---|
| Liver injury | SEC63 | Confusoside | SLC5A2 |
| Liver injury | PKD1 | Confusoside | SLC5A1 |
| Liver injury | TP53 | Confusoside | TYR |
| Liver injury | IL6 | Confusoside | SRD5A1 |
| Liver injury | PRKCSH | Confusoside | SLC5A4 |
| Liver injury | TNF | Confusoside | SLC28A3 |
| Liver injury | PKHD1 | Confusoside | EPHX2 |
| Liver injury | GANAB | Confusoside | AKR1B1 |
| Liver injury | CTNNB1 | Confusoside | CYP19A1 |
| Liver injury | MIR122 | Confusoside | ADORA2A |
| Liver injury | NBAS | Confusoside | PTPN1 |
| Liver injury | MARS1 | Confusoside | SLC29A1 |
| Liver injury | GPT | Confusoside | SLC28A2 |
| Liver injury | ALB | Confusoside | PDE5A |
| Liver injury | PKD2 | Confusoside | PTPN2 |
| Liver injury | PNPLA3 | Confusoside | TDP1 |
| Liver injury | TSC2 | Confusoside | IMPDH1 |
| Liver injury | HFE | Confusoside | MMP1 |
| Liver injury | ABCB11 | Confusoside | MMP7 |
| Liver injury | MIR21 | Confusoside | MMP8 |
| Liver injury | APOE | Confusoside | TOP1 |
| Liver injury | KRT18 | Confusoside | ADORA1 |
| Liver injury | SERPINA1 | Confusoside | CA14 |
| Liver injury | F2 | Confusoside | ADORA2B |
| Liver injury | TERT | Confusoside | IGFBP3 |
| Liver injury | IL10 | Confusoside | TERT |
| Liver injury | NR1H4 | Confusoside | HRAS |
| Liver injury | RINT1 | Confusoside | YARS |
| Liver injury | LRP5 | Confusoside | HSP90AB1 |
| Liver injury | PHKA2 | Confusoside | ADK |
| Liver injury | MET | Confusoside | MGMT |
| Liver injury | EGF | Confusoside | EGFR |
| Liver injury | APC | Confusoside | HCAR2 |
| Liver injury | ALG9 | Confusoside | DNTT |
| Liver injury | MIR34A | Confusoside | ALB |
| Liver injury | ABCB4 | Confusoside | PIK3CA |
| Liver injury | TGFB1 | Confusoside | PIM1 |
| Liver injury | TSC1 | Confusoside | AGTR1 |
| Liver injury | HNF1A | Confusoside | DYRK2 |
| Liver injury | INS | Confusoside | PYGL |
| Liver injury | MIR155 | Confusoside | IGFBP5 |

| | | | |
|---|---|---|---|
| Liver injury | SLC17A5 | Confusoside | MMP9 |
| Liver injury | KRT8 | Confusoside | CSNK2A1 |
| Liver injury | CASP8 | Confusoside | MAPK8 |
| Liver injury | PIK3CA | Confusoside | CASP3 |
| Liver injury | AFP | Confusoside | ESR1 |
| Liver injury | NAFLD1 | Confusoside | PNP |
| Liver injury | CYP2E1 | Confusoside | KDM3A |
| Liver injury | MIR146A | Confusoside | CXCR2 |
| Liver injury | GYS2 | Confusoside | KDM4C |
| Liver injury | FABP1 | Confusoside | ITK |
| Liver injury | IL1B | Confusoside | IRAK4 |
| Liver injury | PPARG | Confusoside | KDM4A |
| Liver injury | PYGL | Confusoside | HSPA8 |
| Liver injury | HADHA | Confusoside | HSPA5 |
| Liver injury | ABCC2 | Confusoside | FYN |
| Liver injury | NAFLD2 | Confusoside | TTR |
| Liver injury | CPT1A | Confusoside | SELE |
| Liver injury | GGT1 | Confusoside | TYMP |
| Liver injury | POLG | Confusoside | BCL2A1 |
| Liver injury | MIR17 | Confusoside | VCP |
| Liver injury | MIR27A | Confusoside | MME |
| Liver injury | ARG1 | Confusoside | PARP1 |
| Liver injury | HNF4A | Confusoside | PTGER1 |
| Liver injury | CYP3A4 | Confusoside | GSK3B |
| Liver injury | CXCL8 | Confusoside | AMPD3 |
| Liver injury | SERPINE1 | Confusoside | CDC25B |
| Liver injury | CRP | Confusoside | RAF1 |
| Liver injury | MIR15A | Confusoside | ATIC |
| Liver injury | FARSB | Confusoside | ALOX12 |
| Liver injury | IGF2R | Confusoside | BRAF |
| Liver injury | UGT1A1 | Confusoside | ITGB1<br>ITGA4 |
| Liver injury | HGF | Confusoside | MARS |
| Liver injury | SLCO1B1 | Confusoside | P2RX3 |
| Liver injury | HMOX1 | Confusoside | AKR1B10 |
| Liver injury | TLR4 | Confusoside | DHODH |
| Liver injury | LARS1 | Confusoside | CDK2<br>CCNA1<br>CCNA2 |
| Liver injury | IFNG | Confusoside | SLC2A1 |
| Liver injury | ATP8B1 | Confusoside | BMP1 |
| Liver injury | GFER | Confusoside | PRKACA |
| Liver injury | SOD1 | Confusoside | LGALS4 |

| | | | |
|---|---|---|---|
| Liver injury | MIR223 | Confusoside | PDE7A |
| Liver injury | AXIN1 | Confusoside | LGALS8 |
| Liver injury | KRAS | Confusoside | NR4A1 |
| Liver injury | HLA-DRB1 | Confusoside | GBA |
| Liver injury | NOS2 | Confusoside | RARS |
| Liver injury | ALG8 | Confusoside | MAPK9 |
| Liver injury | MIR203A | Confusoside | ECE1 |
| Liver injury | ATP7B | Confusoside | LTA4H |
| Liver injury | MIR200B | Confusoside | FOLH1 |
| Liver injury | IGF1 | Confusoside | SCN4A |
| Liver injury | MIR22 | Confusoside | SCN2A |
| Liver injury | CDKN3 | Confusoside | BCL2L2 |
| Liver injury | MIR221 | Confusoside | PPP1CA |
| Liver injury | MIR106B | Confusoside | RNASEH1 |
| Liver injury | MIR181B1 | Confusoside | TYMS |
| Liver injury | APOA1 | Confusoside | PRKCA |
| Liver injury | CPT2 | Confusoside | CHIA |
| Liver injury | G6PC1 | Confusoside | CASP6 |
| Liver injury | MIR143 | Confusoside | CASP7 |
| Liver injury | MIR222 | | |
| Liver injury | CASP3 | | |
| Liver injury | MIR93 | | |
| Liver injury | MIR181A1 | | |
| Liver injury | MIR29A | | |
| Liver injury | SLC25A13 | | |
| Liver injury | MIR25 | | |
| Liver injury | MIR20A | | |
| Liver injury | STAT3 | | |
| Liver injury | FAH | | |
| Liver injury | MMP9 | | |
| Liver injury | HAMP | | |
| Liver injury | PPARA | | |
| Liver injury | TTR | | |
| Liver injury | DNAJB11 | | |
| Liver injury | MIR19A | | |
| Liver injury | EPO | | |
| Liver injury | MIR195 | | |
| Liver injury | ADIPOQ | | |
| Liver injury | NR1H3 | | |
| Liver injury | MAPK1 | | |
| Liver injury | MIR23B | | |
| Liver injury | MIR127 | | |
| Liver injury | F5 | | |

| | | | |
|---|---|---|---|
| Liver injury | MIR181A2 | | |
| Liver injury | TF | | |
| Liver injury | CP | | |
| Liver injury | CYP1A2 | | |
| Liver injury | PDGFRL | | |
| Liver injury | MIR191 | | |
| Liver injury | VEGFA | | |
